# Open source automated insulin delivery: addressing the challenge

**DOI:** 10.1038/s41746-019-0202-1

**Published:** 2019-12-11

**Authors:** Nick Oliver, Monika Reddy, Claire Marriott, Tomas Walker, Lutz Heinemann

**Affiliations:** 1 Division of Diabetes, Endocrinology and Metabolism, Imperial College London, London, UK; 2 Roche Diabetes Care, Burgess Hill, UK; 3 Dexcom, San Diego, CA USA; 4 Science Consulting in Diabetes GmbH, Neuss, Germany

**Keywords:** Type 1 diabetes, Translational research

## Abstract

Do-it-yourself automated insulin delivery systems for people living with type 1 diabetes use commercially available continuous glucose sensors and insulin pumps linked by unregulated open source software. Uptake of these systems is increasing, with growing evidence suggesting that positive glucose outcomes may be feasible. Increasing interest from people living with, or affected by, type 1 diabetes presents challenges to healthcare professionals, device manufacturers and regulators as the legal, governance and risk frameworks for such devices are not defined. We discuss the data, education, policy, technology and medicolegal obstacles to wider implementation of DIY systems and outline the next steps required for a co-ordinated approach to reducing variation in access to a technology that has potential to enable glucose self-management closer to target.

## Introduction

Do-It-Yourself (DIY) automated insulin delivery (AID) solutions use continuous glucose sensors, open source software running on a smartphone, and insulin pumps to create systems that deliver insulin continuously in response to changes in subcutaneous glucose. The algorithm code is available to download from freely available software development platforms such as GitHub which host non-executable code. Implementation of the systems is supported by on-line guides, peer support from expert users and developers, and in some cases, at DIY AID meetings. Increasing uptake of these systems has highlighted the legal, ethical, technical and policy challenges for the diabetes community.

Position statements from Diabetes UK,^1^ Diabetes Australia^2^, and The UK Juvenile Diabetes Research Foundation (JDRF)^3^ have addressed the increasing uptake of “Do-It-Yourself (DIY)” automated insulin delivery (AID) solutions for people living with type 1 diabetes (T1DM). These statements differ slightly in some aspects but are clear that, while people with T1DM using open solutions should have access to appropriate multidisciplinary medical support and education, the systems themselves are not approved medical products that are underpinned by an evidence base for safety or efficacy, and their use renders device warranties invalid. The statements are also clear that open source systems cannot be endorsed by their authors. Concurrently, JDRF is working with partners in industry to enable the development of DIY AID systems^4^ while the rapid pace of recent advancements in diabetes technologies suggests that multiple AID systems will be commercially available in the near future.

From the published reports of selected user cohorts, and from anecdotal evidence, clinically meaningful benefits in glucose outcomes, and reductions in self-management burden are possible with DIY AID systems: HbA1c, time in range (TiR), and improvements in sleep were reported by an early selection of users in 2016,^5^ and an update of the data two years later – including selected reports of national use in Italy and Korea – suggested the same benefits.^6^ Self-reported data of DIY AID users in social media suggest similar improvements in glucose control, diabetes burden and quality of life.^7^ However, these reports lack baseline data and are subject to a considerable selection bias.^8^ A more recent analysis of clinical data from 34 DIY AID users included baseline data using sensor-augmented pump therapy and suggested clinically meaningful improvements in TiR, HbA1c, hypoglycaemia, and glucose variability but were again taken from a small, highly selected patient group.^9^ The Open Project, funded by the European Union, is undertaking further research including the DIWHY Survey into motivations, barriers and retention of DIY AID in the real world.^10^

At present, the clinical governance, regulatory, and medico legal status of DIY AID is unknown. There is no system established for systematic documentation of adverse events and no data are collected appropriately to evaluate safety and efficacy in a broad base of users. For DIY AID, squaring the circle of regulation, governance, medicolegal status, ethics, and personal choice is a novel challenge, and is one that has only recently been enabled by improvements in continuous glucose monitoring (CGM), wireless and processing bandwidth, and insulin pumps.^11^ No single group of professionals or users can address this challenge alone – a collaborative approach is required. In this perspective, we explore some of the ways that a multidisciplinary approach may unlock the potential for open source solutions to be implemented more widely for people with T1DM in a way that is acceptable to all stakeholders, while ensuring sensitivity to the priorities of existing users.

### Data

Wider implementation, support and funding of open source systems for people with T1DM depend wholly on data demonstrating safety and effectiveness. Ideally, health economic evaluation including clear inclusion criteria will also be available in due course. In order to achieve this a robust data collection system must be established to independently collate data at a national, or preferably international level, with associated resources required to achieve this. Data are already voluntarily collected from some users of OpenAPS, but the repository is limited to times when the system is operational and does not include entry (or exit) data^12^ − a prospective dataset must include key glucose and non-glucose outcomes from before and after the use of open source systems. An example of this is the Swedish National Diabetes Register^13^ and it may additionally be feasible to extract data from established electronic health records for users of DIY systems.

An open, rolling data collection system enabling longer term follow-up is critical, along with baseline demographics to enable identification of characteristics most associated with improvements in meaningful outcomes. This necessitates moving beyond early adopters and highly engaged patients to include data more representative of the wider population of people with T1DM. In keeping with the ethos of the open source, community data could potentially be collected remotely from users and healthcare teams as part of usual care. As with all T1DM interventions, understanding the groups of people that receive greatest benefit from DIY AID systems is important and data collection that enables identification of those groups would be powerful. Baseline data collection should therefore extend beyond glycaemic status and basic demographics to include frequency, severity and awareness of hypoglycaemia, psychosocial wellbeing and complication status.

Importantly, any prospective data collection system must also include an adverse event reporting tool, open to users and healthcare teams, with standardised nomenclature for events. An online or anonymous hotline system for users could be established for adverse event reporting and to offer formalised user support. Clinicians involved in the care of people using DIY AID systems should consider that, in many territories, they have a legal obligation to report safety issues to their local health or regulatory authorities. Severe hypoglycaemic events have, without doubt, occurred in people using a DIY AID system, but at present there is no mechanism to report this. A recent FDA notice was sent warning of an adverse event which had been reported while using a DIY AID system.^14^ While anecdotal reports exist, the lack of a formal reporting system remains a legitimate concern regarding these systems. Finally, open publication of the data must be built in to the system, ideally in real-time or near real-time, as with the Open Prescribing reporting system,^15^ ensuring users and stakeholders are able to view aggregated data in a transparent way.

### Education

Diabetes technology is an adjunct to support and education, and its impact on glucose outcomes is dependent on self-management actions. It is therefore critical in a DIY AID implementation that appropriate education is provided, both to users (where allowable by the medicolegal framework), and to healthcare professionals.

As with all T1DM interventions, an in-depth understanding of the clinical impact, and mechanism of action of DIY AID, is important for healthcare professionals. Previous models of healthcare professional education, dependent on experience gained in clinical scenarios may not be sufficient for technologies used by a small number of people. For effective implementation, centres of expertise may be required to deliver services, and to train healthcare professionals to understand, and to appropriately support, DIY AID in a hub-and-spoke model. Integrated automated solutions will, over time, become standard of care but safe implementation, as with all invasive procedures, requires familiarity and frequent exposure. Healthcare professional expertise can be a limiting factor in technology uptake^16,17^ and expertise in established technologies may need to be addressed before DIY AID systems can be widely supported.

As with insulin pump therapy, CGM, and insulin pen usage, user and caregiver education is critical to ensure safe and effective use. Healthcare professionals spend a relatively small amount of time with people living with T1DM meaning that achieving optimal outcomes is dependent on empowering people to make treatment decisions in real time and not only at each clinical interaction. Using DIY AID requires an in-depth understanding of the settings which include basal rates, insulin: carbohydrate ratio, insulin sensitivity factor, and insulin on board. These concepts are included in evidence based structured education programmes^18^ but may require refreshment, as well as support for more complex and adaptable settings and additional tools to enable dynamic changes. At present, a blended model of user education exists with the responsibility for structured education resting with clinical teams and automated insulin delivery support provided by peers. Delivery of structured education is supported by international standards of care^19–22^ and is a key component of a T1DM clinical service – its importance is not diminished by AID, but is likely to be enhanced to ensure maximal value is extracted from a complex ecosytem. The ability of the user to understand and adjust the parameters of their open-source platform should be considered when assessing a system’s suitability. Structured education programmes will need to be adapted over time to meet the needs of AID, as has occurred with the evolution of the DAFNE programme to include pump therapy and hypoglycaemia awareness training.^23^ Creation of an AID specific education curriculum, based on evidence-informed training such as DAFNE would enable support and education to be delivered with quality assurance and auditable standards.

### Policy

Widespread implementation of any intervention requires supportive policy to ensure equitable access in line with the evidence base for clinical, and cost, effectiveness. Parallel to frequently reported clinical outcomes, DIY AID systems require policy to assess prioritisation and funding.

At present, open source systems are dependent on a combination of health service, insurer, and personal funding for an insulin pump, insulin pump consumables, CGM system, and insulin. Data for the payer funded contribution to open systems is not available and payer support for commercially available (hybrid) AID systems (for example the Medtronic 670G^24^) is dependent on local contracting arrangements. This blended funding model, along with an absence of high quality evidence for glucose outcomes and adverse events, makes assessment of the health economic case for DIY AID systems challenging. Generation of larger, more comprehensive datasets, especially if linked to healthcare utilisation through electronic health records, would enable estimation of the cost effectiveness case for DIY systems which would support policy and regulation.

Previous reports have highlighted variation in access to technologies.^25^ Evidence-based policy to underpin commissioning of systems for people likely to derive clinically important benefit is critical to wider equitable adoption of an open architecture, with funding transparently attached to that. Independent review through health technology appraisal bodies such as the European Network for Health Technology Assessment would then be feasible, providing robust policy support.

### Ethical and legal responsibility

The ethical, legal and governance structures underlying DIY AID systems are, at present, unknown and complex, with local variance. To enable equitable access for people living with T1DM, a multi-agency approach is required to assess the risks, and to define clinician, regulatory and manufacturer responsibility and accountability. This needs people with T1DM, health services, lawyers, payers, indemnity and defence organisations, regulators, and industry representation (from device and pharmaceutical manufacturers) to agree a clear governance framework.

In order to facilitate this there is an additional responsibility on the creators of DIY AID systems to seek peer-review of algorithms and interventions. As with any T1DM treatment, peer-review ensures transparency, a referenceable standard, reproducible data, and clarity of expected outcomes. It also allows others to objectively appraise it. In a technical architecture where software versions may be frequently updated, the traditional model of testing, evaluation and publication may not be fit for purpose and newer publication models, such as the open science repository model,^26,27^ may be more appropriate, with a master framework published in a peer-reviewed format. All published algorithms should share a pre-defined validation metric such as standardised in silico^28^ testing to enable cross-comparison.

A legal evaluation of DIY AID systems was undertaken in Germany (commissioned by the German Diabetes Association), exploring the medical, criminal and civil law implications in July 2018.^29^ The detailed response included the following recommendations:

- The assembly of a DIY AID system is not a criminal offence. However, as the intended purpose of the devices is violated, there is no liability on the part of the manufacturers of the medical devices.
- People who build DIY AID systems and transfer them to other patients are liable to prosecution under the Medical Devices Act in Germany. The placing on the market, and commissioning of such a system are prohibited. The person who builds and transfers the system is responsible under the Product Liability Act.
- Healthcare professionals do not have to refer people with T1DM to open source systems.
- If a person with T1DM expresses interest in such a system or is already using it, the healthcare professional must inform the patient of the improper use of a medical device and of the associated risks, documenting this appropriately.
- Healthcare professionals should not offer a platform for exchange of DIY AID systems or training - this may be viewed as an application of a medical device contrary to its intended purpose.
- If a healthcare professional trains people to use DIY AID systems, they become the operator of a medical device with potential liability consequences under the Medical Devices Act.
- If a healthcare professional links DIY AID user experts with people with T1DM, they may be prosecuted for placing medical devices on the market without CE marking.
- If medical devices are used contrary to their intended purpose, healthcare professionals are not liable to prosecution under the Medical Devices Act. Under certain conditions, however, criminal liability may be considered for negligent killing or negligent injury.
- Healthcare professionals are obliged to inform users about the intended use of medical devices.
- Healthcare professionals must point out the dangers that may arise when using a DIY AID system and should clearly distance themselves from the use of an open system and not encourage patients to use the system.

This legal review is only applicable in Germany, but is an excellent example of providing medicolegal clarity to developers, users and healthcare professionals and serves as a model for other territories to follow.

It is also important to consider the insulin used in AID systems and whether present licensing and prescribing is appropriate. Most insulin prescriptions are completed by primary care and clarity over prescribing in line with local licensing may be required to ensure prescriptions for AID meet local prescribing standards. While governance for prescription, dispensing and use of insulin is held by the healthcare professional, pharmacist and user, there may be additional pharmacovigilance responsibilities for insulin manufacturers which cannot be met when insulin is used in unlicensed systems, and inclusion of insulin manufacturers as a stakeholder in the development of DIY AID reporting systems is critical.

### Technology

The United States Food and Drugs Administration (FDA) has introduced the interoperable CGM (iCGM)^30^ and alternate controller enabled (ACE) pump standards^31^ which include accuracy and interoperability standards for future automated insulin delivery. At the time of writing, one CGM system and two insulin pumps meet those special measures, and no interoperability standards for a controller have been defined, though it is likely that a controller standard will be created. International work to replicate this may enable open source systems to be available alongside others with an identical level of regulation and, where allowed by manufacturers, for them to be integrated with off-the-shelf components. Currently, some open source systems rely on legacy insulin pumps that are no longer supported, and unauthorized software to link components which were never designed to be compatible. Vulnerabilities in legacy insulin pumps have been highlighted by cyber security experts,^32^ leading to a security bulletin for some devices by the manufacturer.^33^ Older equipment and unlicensed software solutions should be replaced with modern equipment and validated communications to support a safer underlying framework for open source systems.

However, while interventions and regulatory standards may mitigate some future challenges, personal choice and availability may still drive open source innovation, and systems may continue to sit outside of enhanced standards of interoperability. Currently, manufacturers of interoperable systems remain more focused on commercial partnerships than on open source development. There is, however, some exploration into how these pathways could be leveraged by the open source community.^34^ How these systems are classified and regulated will remain a challenge to be addressed and, while the FDA model is one to aspire to and has received advocacy and commercial support, it does not wholly address the issue of DIY AID, and is only applicable in the United States. Implementation of a similar system or wider adoption of the FDA model internationally would accelerate access to all AID systems and would enable more rapid data collection to demonstrate clinical and cost effectiveness.

### Future Steps

AID remains a target for people with T1DM, for healthcare professionals, and for the medical device industry. Open source innovation has driven increased uptake of AID solutions, but implementation challenges exist throughout the pathway, with undefined risks to users, healthcare professionals and commercial stakeholders. A multidisciplinary approach including an assessment of regulatory hurdles is required to solve these challenges for all stakeholders. In order to progress the following are required:

1. Creation of an open access prospective data collection tool that includes adverse event reporting and is available to users and healthcare teams. Endpoints should include overall glycaemia (measured by time spent in range 3.9 to 10 mmol/L as well as HbA1c);^35^ time spent in hypoglycaemia (<3.0 mmol/L);^36^ the frequency of severe hypoglycaemia; hypoglycaemia awareness and fear; a measure of self-management confidence; quality of life, and a measure of diabetes distress as a minimum. Inclusion of data collected before initiation of AID is critical.
2. Comprehensive data entry by users and healthcare teams, including those not regularly interacting with a specialist team. Data entry must include positive and negative experiences, as well as by those that discontinue DIY AID.
3. Frequent data reporting from the longitudinal dataset which must include potential safety signals reported in the MHRA,^37^ EUDAMED,^38^ and MAUDE data bases.^39^
4. A peer-reviewed framework for all algorithms, including a standardised in silico dataset.
5. Establishment of specialist AID centres to provide quality assured healthcare professional and patient education in a hub and spoke model.
6. Dynamic central policy that reflects the evidence base for clinical, and cost, effectiveness and is linked to funding.
7. A multi-agency approach to regulation, ideally starting immediately and including an independent legal review, with clearly defined goals and responsibilities to address interoperability standards and to manage systems that operate outside of those standards. Efforts at a national and international level will be required with devolved leadership groups in each territory.

## Conclusion

The progression of DIY AID is a shared responsibility and significant collaborative efforts are required to progress regulation, publication, and data generation sufficiently to enable equitable access for people with T1DM, and to enable the potential benefits of technologies to be realised at scale. The proposed actions are not comprehensive and will not solve all issues associated with DIY AID but may serve as a blueprint to begin squaring the circle.

## Data Availability

No datasets were generated or analysed during the current study.
